# The effects of 3D culture on the expansion and maintenance of nucleus pulposus progenitor cell multipotency

**DOI:** 10.1002/jsp2.1131

**Published:** 2020-12-08

**Authors:** Julien Guerrero, Sonja Häckel, Andreas S. Croft, Christoph E. Albers, Benjamin Gantenbein

**Affiliations:** ^1^ Tissue Engineering for Orthopaedics & Mechanobiology, Department for BioMedical Research (DBMR) of the Faculty of Medicine of the University of Bern University of Bern Switzerland; ^2^ Department of Orthopaedic Surgery & Traumatology, Inselspital Bern University Hospital Bern Switzerland

**Keywords:** adipogenesis, alginate bead, angiopoietin‐1 receptor, chondrogenesis, differentiation, flow cytometry, intervertebral disk, microenvironment, nucleus pulposus, osteogenesis, qPCR, three‐dimensional culture, tissue‐specific progenitor cells

## Abstract

**Introduction:**

Low back pain (LBP) is a global health concern. Increasing evidence implicates intervertebral disk (IVD) degeneration as a major contributor. In this respect, tissue‐specific progenitors may play a crucial role in tissue regeneration, as these cells are perfectly adapted to their niche. Recently, a novel progenitor cell population was described in the nucleus pulposus (NP) that is positive for Tie2 marker. These cells have self‐renewal capacity and *in vitro* multipotency potential. However, extremely low numbers of the NP progenitors limit the feasibility of cell therapy strategies.

**Objective:**

Here, we studied the influence of the culture method and of the microenvironment on the proliferation rate and the differentiation potential of human NP progenitors *in vitro*.

**Method:**

Cells were obtained from human NP tissue from trauma patients. Briefly, the NP tissue cells were cultured in two‐dimensional (2D) (monolayer) or three‐dimensional (3D) (alginate beads) conditions. After 1 week, cells from 2D or 3D culture were expanded on fibronectin‐coated flasks. Subsequently, expanded NP cells were then characterized by cytometry and tri‐lineage differentiation, which was analyzed by qPCR and histology. Moreover, experiments using Tie2^+^ and Tie2^−^ NP cells were also performed.

**Results:**

The present study aims to demonstrate that 3D expansion of NP cells better preserves the Tie2^+^ cell populations and increases the chondrogenic and osteogenic differentiation potential compared to 2D expansion. Moreover, the cell sorting experiments reveal that only Tie2^+^ cells were able to maintain the pluripotent gene expression if cultured in 3D within alginate beads. Therefore, our results highly suggest that the maintenance of the cell's multipotency is mainly, but not exclusively, due to the higher presence of Tie2^+^ cells due to 3D culture.

**Conclusion:**

This project not only might have a scientific impact by evaluating the influence of a two‐step expansion protocol on the functionality of NP progenitors, but it could also lead to an innovative clinical approach.

## Graphical abstract

The present study aims to demonstrate that 3D expansion of NP cells better preserves the Tie2+ cell populations and increases the chondrogenic and osteogenic differentiation potential compared to 2D expansion. Our results highly suggest that the maintenance of multipotent capacity is mainly but not exclusively due to the higher presence of Tie2+ cells due to 3D culture.

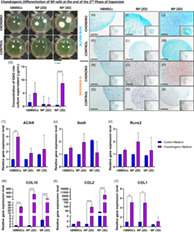

## 1 INTRODUCTION

The intervertebral disk (IVD) consists of superior and inferior cartilaginous endplates (CEP), outer annulus fibrosus (AF), and central gel‐like nucleus pulposus (NP). IVD degeneration is a major cause of lower back pain, and the burdens caused by IVD degeneration‐related morbidities extend to society and the economy as a whole.^1^, ^2^, ^3^, ^4^ Current therapies cannot cure IVD degeneration and are limited to conservative treatments (such as medication or physical therapy) or surgical interventions (such as spinal fusion).^5^ Regeneration therapy for IVD degeneration mainly targets the NP tissue, as its degeneration is considered to be a major cause of low back pain.^5^, ^6^ Therefore, understanding the cell morphology and gene expression profile of NP cells (NPCs) is critical for developing new therapeutic strategies for IVD regeneration.

The recent discovery of a new cell candidate for cell therapy, the NP progenitor cell (NPPC), advances this research focus as this cell type might be involved in the innate maintenance of disk health.^7^ These progenitor cells represent a rare subpopulation (between 1% and 5%) of the total NP cell population and were shown to decrease markedly with age and to be nearly absent in people >40 years of age.^7^ However, NPPCs fulfilled formally progenitor criteria and possess the ability for multilineage differentiation.^7^, ^8^ Such NPPCs were identified and successfully isolated from human, bovine, murine, and canine NP tissues using fluorescence‐activated cell sorting (FACS) through the angiopoietin‐1 receptor (aka. Tie2).^9^ Nonetheless, it is still to be clarified how this Tie2 positive (Tie2^+^) cells are related to mesenchymal stromal cells (MSCs) and the recently closer characterized notochordal cells.^10^ Recently, a study reported the expression of two classical MSC markers, CD29 and CD105, in NPPCs compared to umbilical‐cord‐derived MSCs.^11^ These results were in accordance with findings of niches for multipotent stem cells in the disk itself or possibly in the nearby bone marrow of the vertebrae close to CEP cells.^12^, ^13^, ^14^

The ability to culture and expand NPCs (and especially NPPCs), while maintaining their gene expression profile and their stem potential is essential in experiments using these cells, as dysregulation of multiple intracellular signaling pathways may play a key role in IVD degeneration.^15^, ^16^ Several cell based or cytokine therapies have been investigated using rat, rabbit, bovine, mouse, and human NPC culture,^6^, ^17^, ^18^, ^19^ but molecular pathways such as NPC intracellular signaling pathways are still not fully understood.^1^, ^5^

Cell therapy seems to be a more promising approach for disk regeneration. However, so far, there have been few reports using human NPCs caused by the difficulty associated with harvesting adequate quantities of these cells from available IVDs. Additionally, despite their early fading, the study of this marginal subpopulation within the NP might give rise to a better understanding of how these cells affect IVD health, and what conditions will be optimal to extend their number in vitro and to maintain them longer in their native stem state. Moreover, a deeper understanding might lead to new approaches for the regeneration of the IVD. Furthermore, the gene expression profile of human NPCs is easily lost with conventional two‐dimensional (2D) monolayer culture, which does not replicate the three‐dimensional (3D) hydrophilic aggrecan‐rich gelatinous extracellular matrix (ECM) with unique avascular, hypoxic, nutrient‐deficient, high osmotic pressure characteristics in which NPCs are naturally supported. Therefore, a novel culture method is required, which allows for the number of NPCs to be expanded while maintaining their gene expression profile. In this study, we aimed to establish an in vitro culture method for human NPCs to maintain and increase the amount of NPPCs over time. We also aimed to preserve the stem potential of those cells regarding their multipotent gene expression and phenotype but also their ability to differentiate into a chondrogenic, adipogenic, and osteogenic lineage. Moreover, experiments were performed in which NPCs were sorted into Tie2^+^ and Tie2^−^ subpopulations combined with our new culture method was also performed to better understand the metabolism and gene expression profile of those cells under those specific conditions.

## 2 MATERIALS AND METHODS

### 2.1 Cell isolation

Human IVD tissue was obtained from 10 patients ranging from 20 to 70 years of age (mean ± SD [SD]) (36.81 ± 15.61 years) undergoing spinal surgery after experiencing trauma to their disks. Immediately after harvesting the IVDs, an experienced surgeon divided the tissues into annulus fibrosus (AF), nucleus pulposus (NP), and cartilaginous endplate (CEP). The tissues were subsequently processed in the laboratory within 24 hours after surgery. Patients provided written consent and the ethics committee of the Canton of Bern approved the procedure (SwissEthics # 2019‐00097).

First, the human disk cells were isolated from their native extracellular matrix (ECM) by sequential digestion of the tissues with 1.9 mg/mL Pronase (Roche Diagnostics, Mannheim, Germany) for 1 hour and then with Collagenase type II (Worthington, London, UK) at a concentration corresponding to the digested tissue (NP: 64 U/mL, AF: 129 U/mL, CEP: 1562 U/mL per gram tissue) on a plate shaker at 10 RPM at 37°C, overnight. Remaining tissue fragments were removed by using filtration through a 100 μm cell strainer (Falcon, Corning, New York). Right after isolation, NP cells were expanded separately in the proliferation medium consisting of low‐glucose (1 g/L) Dulbecco's Modified Eagle Medium (LG‐DMEM; Gibco, Life Technologies, Zug, Switzerland), supplemented with 10% fetal bovine serum (FBS) and 1% penicillin/streptomycin/glutamine (#10378016; 10  000 units of penicillin, 10  000 μg of streptomycin, and 29.2 mg/mL of l‐glutamine in a 10 mM citrate buffer; Merck, Darmstadt, Germany). The AF and CEP cells were directly frozen and stored right after isolation.

Concerning human bone marrow mesenchymal stromal cells (hBMSCs), human bone marrow aspirates (20 mL) were obtained during routine orthopedic surgical procedures on the same patients described above undergoing spinal surgery after experiencing trauma to their disks. After surgery, the bone marrow aspirate was immediately transferred into plastic tubes containing heparin, S‐Monovette (#01.1634; Sarstedt, Nümbrecht, Germany). After diluting the marrow aspirates with phosphate‐buffered saline (PBS) at a ratio of 1:4, nucleated cells were isolated using a density gradient solution (Histopaque, Sigma). The complete medium consisted of α‐minimum essential medium (αMEM) with 10% fetal bovine serum, 1% of 4‐(2‐hydroxyethyl)‐1‐piperazineethanesulfonic acid (aka. HEPES) at 1 M, 1% sodium pyruvate (100 mM), and 1% of penicillin‐streptomycine‐glutamine (100×) solution (P/S/G) (from Gibco and Merck, respectively). Nucleated cells were plated at a density of 3.10^6^ cells/cm^2^ in complete medium supplemented with 5 ng/mL of fibroblast growth factor‐2 (FGF‐2; R&D Systems, Zug, Switzerland) and cultured in a humidified incubator at 37°C and 5% CO_2_. The medium was changed twice a week. Here, hBMSCs were selected based on adhesion and proliferation on the plastic substrate 1 week after seeding and were used at passage 3.

### 2.2 Cell culture

#### 2.2.1 Conventional 2D monolayer culture of human nucleus pulposus cells

For the 2D culture in the first expansion phase, NP cells isolated from IVDs were plated at 1 × 10^4^ cells/cm^2^ on culture flask/dish (Techno Plastic Products, Trasadingen, Switzerland) and cultured in LG‐DMEM with 10% fetal bovine serum, 1% HEPES (1 M), 1% sodium pyruvate (100 mM), and 1% of PSG (100×) solution (all from Gibco) and 100 μM l‐ascorbic acid‐2‐phosphate (Sigma‐Aldrich) under a 5% CO_2_ and 5% oxygen atmosphere at 37°C. The medium was replaced twice a week until confluence.

#### 2.2.2 Primary 3D culture of human nucleus pulposus cells in alginate beads

For the 3D culture in the first expansion phase, NP cells isolated from NP tissue were washed with PBS and centrifuged at 1500 rpm for 3 minutes. Cells were mixed and encapsulated in 1.2% alginate at a density of 4 × 10^6^ cells/mL using a syringe (22 G needle) by forming approximately 30 μL droplets in a 102 mM CaCl_2_ salt solution.^20^ The NP cell alginate beads (six beads per well of 12‐well plate) were maintained in LG‐DMEM with 10% fetal bovine serum, 1% HEPES (1 M), 1% sodium pyruvate (100 mM), and 1% of penicillin‐streptomycin‐glutamine (100×) solution (all from Gibco) and 100 μM l‐ascorbic acid‐2‐phosphate (Sigma‐Aldrich) under a 5% CO_2_ and 5% oxygen atmosphere at 37°C, for 7 days. The medium was replaced twice a week.

#### 2.2.3 For the second phase of expansion in 2D monolayer for human nucleus pulposus cells

After 7 days of primary 2D conventional monolayer culture (first expansion phase), NPCs were isolated from tissue culture dishes by trypsinization, washed, and seeded back into tissue culture dishes coated with fibronectin (#F2006, Sigma) at a density of 10 × 10^3^ cells/cm^2^. Thereafter, 3D NP cell alginate beads were digested in alginate dissociation buffer, composed of NaCl at 150 mM (#71379, Sigma) and Na_3_Citrate.2H_2_O at 55 mM (#71406, Sigma) dissolved in Milli‐Q‐Water and adjusted at pH = 6.8, at 37°C for 15 minutes. After washing, NPCs were seeded into a tissue culture dish coated with fibronectin (#F2006, Sigma) at a density of 10 × 10^3^ cells/cm^2^ with LG‐DMEM with 10% fetal bovine serum, 1% HEPES (1 M), 1% sodium pyruvate (100 mM), and 1% of penicillin‐streptomycin‐glutamine (100×) solution (all from Gibco) and 10 ng/mL of FGF‐2 (R&D Systems) until confluence. After reaching confluence, cells were directly used for the differentiation assays. Microscopic pictures were taken for each condition at the end of the first and second phase of expansion using the Nikon Eclipse E800 microscope (Nikon, Tokyo, Japan).

### 2.3 FACS analysis

The phenotype of NPCs was determined by cytofluorimetric analysis with fluorochrome‐conjugated antibodies against human CD90‐FITC (#555595) at 1:100, CD73‐PE (#550257) at 1:100, CD105‐Alexa488 (#MHCD10520) at 1:100, CD34‐PECy5 (#555823) at 1:100, CD45‐PECy7 (#557748) at 1:100, CD146‐BV605 (#7433019) at 1:100, and Tie2‐PE (#FAB3131A) at 1:50 (all from BD Bioscience, Franklin Lakes, NJ). Isotype IgG was used as a control (all from BD Biosciences). Cells in suspension were incubated for 40 minutes with each of these antibodies at 4°C in FACS buffer (PBS, 0.5% human serum albumin, 0.5 mm EDTA), and analyzed with a Cell Lab Quanta SC flow cytometer (Beckman Coulter Inc.).

### 2.4 Adipogenic differentiation

For adipogenic differentiation, NPCs were seeded at a density of 15 × 10^3^ cells/cm^2^ in a 12‐well plate (Techno Plastics Products, Inc.). Cells were grown until 90% confluency was reached in monolayer culture in α‐MEM supplemented with 10% FBS, Sigma‐Aldrich, 1% penicillin/streptomycin (P/S, 100  μg/mL and 100 IU/mL, respectively; Merck), and 2.5  ng/mL fibroblast growth factor 2 (FGF2; #100‐18B; PeproTech, London, UK). The medium was then changed to adipogenic differentiation medium (α‐MEM with 10% FCS, 1% P/S, 12.5  μM insulin, 100  nM dexamethasone, 0.5 mM 3‐isobutyl‐1‐methylxanthine, and 60 μM indomethacin [all from Sigma‐Aldrich]) and incubated for 21 days under a 5% CO_2_ and 20% oxygen atmosphere at 37°C.^21^ NPCs that were kept in complete medium (α‐MEM supplemented with 10% FBS, Sigma‐Aldrich, 1% penicillin/streptomycin [P/S, 100 μg/mL and 100 IU/mL, respectively; Merck ]) were used as control. The medium was changed twice a week, and samples were carried out in technical duplicates (ie, in two wells).

Adipogenic differentiation was evaluated by light microscopy after staining the lipid vacuoles with Oil Red O (Merck). Samples were fixed with 4% formaldehyde; the cell layer was rinsed with 50% EtOH and stained for 20  minutes with 0.2% (w/v) Oil Red O in 60% (v/v) 2‐propanol (Sigma‐Aldrich). The lipid droplet formation was then visualized under a stereomicroscope (Nikon Eclipse E800) and photographed. For quantification purposes, the Oil Red O was also extracted with 100% 2‐propanol and then analyzed by measuring the absorbance at 500  nm with the microplate reader SpectraMax M5 (Molecular Devices, San Jose, California, distributed by Bucher Biotec, Basel, Switzerland).

### 2.5 Osteogenic differentiation

For osteogenic differentiation, NPCs were seeded at a density of 15 × 10^3^ cells/cm^2^ in a 12‐well plate (Techno Plastics Products, Inc.). Cells were grown until 90% confluency was reached in monolayer culture using an expansion medium (α‐MEM supplemented with 10% FBS, Sigma‐Aldrich, 1% penicillin/streptomycin [P/S, 100  μg/mL and 100 IU/mL, respectively; Merck ], and 2.5 ng/mL fibroblast growth factor 2 [FGF2; #100‐18B; PeproTech]). For differentiation, the medium was changed to an osteogenic medium that consisted of α‐MEM supplemented with 1% P/S, 10% FCS, 100 nM dexamethasone, 10  mM β‐glycerophosphate, and 50  μM l‐ascorbic acid‐2‐phosphate (all from Sigma‐Aldrich).^22^ NPCs in complete medium (α‐MEM supplemented with 10% FBS, Sigma‐Aldrich, 1% penicillin/streptomycin [P/S, 100 μg/mL and 100 IU/mL, respectively; Merck ]), seeded at the same density were used as control. The culture was maintained for 21 days under a 5% CO_2_ and 20% oxygen atmosphere at 37°C, and the medium was refreshed twice a week. The differentiation assay was carried out with two technical duplicates.

The cells were fixed in 4% formaldehyde, and the calcium deposition of the cell layers was evaluated with 2% Alizarin red staining (ALZR) solution (Sigma‐Aldrich) for 45  minutes. The ALZR was released from the cell layers by the addition of a 10% cetylpyridinium chloride solution (Sigma‐Aldrich) for 1 hour under agitation. Optical density was measured at 570 nm with the microplate reader SpectraMax M5.

### 2.6 Chondrogenic differentiation

For chondrogenic differentiation, 300 000 NP cells were seeded as a cell pellet into 1.5 mL Eppendorf tubes for 21 days in chondrogenic medium (HG‐DMEM supplemented with 1% Pen/Strep, 100 nM dexamethasone, 1% insulin‐transferrin‐sodium selenite [ITS+, cat. no. I2521; Sigma‐Aldrich], 1% nonessential amino acids (Gibco, Thermo Fisher Scientific, Basel, Switzerland), 250  μM ascorbic acid, 10  ng/mL transforming growth factor‐beta 3 [TGF‐β3; Peprotech]) or in control medium (serum‐free medium,ie, HG‐DMEM supplemented with ITS+ with 1% P/S), and cultured for 21 days.^23^, ^24^ The medium was changed twice a week, and samples were carried out with biological duplicates. The formed pellets were fixed in 4% formaldehyde for overnight and then embedded in paraffin. Thereafter, 6  μm thin sections were cut with a microtome (Microm HM355; Thermo Fisher Scientific).

The sample sections were stained with 0.02% Fast Green solution (Merck) for 10  minutes. Then they were rinsed with 1% acetic acid and then immersed in 0.1% Safranin‐O (Merck) at pH 2.5 for 15  minutes to stain sulfated glycosaminoglycans (GAGs). Additionally, sections were stained for sulfated GAGs with 1% Alcian blue 8GX (Sigma‐Aldrich) in 3% acetic acid at pH 1.0.^25^ Once dehydrated and mounted, slides were imaged using an inverted microscope (Eclipse E800; Nikon), and single images were taken at ×4 magnification and stitched together using NIS Elements microscope imaging software (Nikon) and edited for easier comparison with ImageJ 1.51j8 (Rasband WS, 2020, ImageJ, National Institutes of Health, Bethesda, Maryland, http://rsb.info.nih.gov/ij/).

### 2.7 Cell sorting

Tie2^+^ cells were sorted using FACS as previously described.^8^, ^9^, ^26^ Freshly isolated NPCs (N = 3) were incubated with an anti‐human Tie2 PE‐conjugated monoclonal mouse antibody (#FAB3131P, clone 83 715, R&D systems) for 30  minutes on ice in 100  μL of FACS buffer ([PBS] with 0.5% bovine serum albumin [BSA] and 1 mM EDTA, all from Sigma‐Aldrich), protected from light. Propidium iodide (Sigma‐Aldrich) was used to exclude dead cells. Cell sorting was performed by FACS Diva III (BD Biosciences) based on the procedure previously reported.^26^ The Mouse IgG_1_ PE‐conjugated Antibody (#IC002P, clone 11 711, R&D systems) was used as isotype control to set the gate for sorting.

### 2.8 Qantitative polymerase chain reaction

Concerning the analysis of 2D vs 3D culture during the second phase of expansion, the gene expression of several major pluripotent genes (homeobox protein NANOG [*NANOG*], octamer‐binding transcription factor 4 [*OCT4*], sex‐determining region Y‐box 2 [*SOX2*]) was analyzed using quantitative polymerase chain reaction (qPCR).

For the three differentiation assays, adipogenic related gene expression (peroxisome proliferator‐activated receptor gamma [*PPARγ*], CCAAT/enhancer‐binding protein alpha [*CEBPα*], and adiponectin [*ADIPOQ*]), osteogenic related gene expression collagen type I [*COL1*], Runt‐related transcription factor 2 [*RUNX2*], alkaline phosphatase [*ALPL*], osterix [*SP7*], osteocalcin [*OCN*] and osteopontin [*OPN*]) and chondrogenic related gene expression (aggrecan [*ACAN*], collagen type II [*COL2*], collagen type X [*COL10*], transcription factor SOX‐9 [*SOX2*], and transforming growth factor‐beta 1 [*TGF‐β1*]) were analyzed using quantitative polymerase chain reaction (qPCR).

RNA isolation was performed as previously described.^27^ DNase (DNase 1 Kit, Sigma‐Aldrich) was used to degrade residual DNA, and iScript cDNA Synthesis Kit (Bio‐Rad Inc., Switzerland) was used for reverse transcription. Thereafter, the cDNA was mixed with iTaq universal SYBR Green Supermix (Bio‐Rad) and with forward and reverse primers (Microsynth, Switzerland) for each gene (Table 1). qPCR was performed in duplicates (CFX96 Touch, Bio‐Rad) using 18S and GAPDH as reference genes. The relative gene expression was determined using the 2^−ΔΔCt^ method,^28^ and data were normalized to the control condition (hBMSCs in complete medium or NP cells in 2D culture).

**TABLE 1:** Overview of all genes used for RT‐Q‐PCR in this study

Gene	Description	Forward primer (5′‐3′)	Reverse primer (3′‐5′)
Reference genes			
18S	18S ribosomal RNA	CGA TGC GGC GGC GTT ATT C	TCT GTC AAT CCT GTC CGT GTC C
GAPDH	Glyceraldehyde 3‐phosphate dehydrogenase	ATC TTC CAG GAG CGA GAT	GGA GGC ATT GCT GAT GAT
Pluripotent‐related genes			
NANOG	Nanog homeobox	CCG AAG AAT AGC AAT GGT	CTG GTG GTA GGA AGA GTA
OCT4	Octamer‐binding transcription factor 4	CGA TCA AGC AGC GAC TAT G	GCC AGA GGA AAG GAC ACT
SOX2	SRY (sex determining region Y)‐box 2	AGA GAG AAA GAA AGG GAG AGA A	GCC GCC GAT GAT TGT TAT
TEK	Angiopoietin‐1 receptor (Tie2)	TTA GCC AGC TTA GTT CTC TGT GG	AGC ATC AGA TAC AAG AGG TAG GG
Adipogenic lineage‐related genes			
PPARγ	Peroxisome proliferator‐activated receptor gamma	ACG AAG ACA TTC CAT TCA CAA GA	CTC CAC AGA CAC GAC ATT CAA
CEPBα	CCAAT/enhancer‐binding protein alpha	CAA GAA CAG CAA CGA GTA	GTC ATT GTC ACT GGT CAG
ADIPOQ	Adiponectin	CCG TGA TGG CAG AGA TGG	TAT ACA TAG GCA CCT TCT CCA G
Chondrogenic lineage‐related genes			
SOX9	Transcription factor SOX‐9	GAG ACT TCT GAA CGA GAG	GGC TGG TAC TTG TAA TCC
TGF‐β1	Transforming growth factor‐beta 1	CGT GCT AAT GGT GGA AAC	GCT CTG ATG TGT TGA AGA AC
COL2	Collagen type 2 A1	AGC AGC AAG AGC AAG GAG AA	GTA GGA AGG TCA TCT GGA
COL10	Collagen type 10 A1	GAA TGC CTG TGT CTG CTT	TCA TAA TGC TGT TGC CTG TTA
ACAN	Aggrecan	CAT CAC TGC AGC TGT CAC	AGC AGC ACT ACC TCC TTC
Osteogenic lineage‐related genes			
ALPL	Alkaline phosphatase	CCT TCA CTG CCA TCC TGT A	CGC CTG GTA GTT GTT GTG
RUNX2	Runt‐related transcription factor 2	AGC AGC ACT CCA TAT CTC T	TTC CAT CAG CGT CAA CAC
OCN	Osteocalcin	GCA GAG TCC AGC AAA GGT G	CCA GCC ATT GAT ACA GGT AGC
OPN	Osteopontin	ACG CCG ACC AAG GAA AAC TC	GTC CAT AAA CCA CAC TAT CAC CTC G
COL1	Collagen type 1 A2	GTG GCA GTG ATG GAA GTG	CAC CAG TAA GGC CGT TTG
SP7	Osterix	CAG GCT ATG CTA ATG ATT ACC	GGC AGA CAG TCA GAA GAG

*Note:* As reference genes: *18S* (18S ribosomal RNA) and *GAPDH* (glyceraldehyde 3‐phosphate dehydrogenase). Genes analyzed for pluripotent markers: *NANOG* (nanog homeobox), *OCT4* (octamer‐binding transcription factor 4), *SOX2* (Sex determining region Y‐box 2), and *TEK* (angiopoietin‐1 receptor). Genes analyzed for the adipogenic lineage: *PPARγ* (peroxisome proliferator‐activated receptor gamma), *CEBPα* (CCAAT/enhancer‐binding protein alpha), and *ADIPOQ* (adiponectin). Genes analyzed for the chondrogenic lineage: *SOX9* (transcription factor Sox‐9), *TGF‐β1* (transforming growth factor‐beta 1), *COL2* (collagen type II), *COL10* (collagen type X), and *ACAN* (Aggrecan). Genes analyzed for the osteogenic lineage: *ALPL* (alkaline phosphatase), *RUNX2* (runt‐related transcription factor 2), *OCN* (osteocalcin), *OPN* (osteopontin), *COL1* (collagen type I), and *SP7* (Osterix).

### 2.9 Cell activity

Mitochondrial activity was determined with the Alamar Blue assay using 50 μM resazurin sodium salt (Sigma‐Aldrich, Switzerland). Samples were incubated with the complete medium containing 10% of 50 μM resazurin solution for 3 hours before reading relative fluorescence units (RFUs) at an excitation wavelength of 547 nm and an emission wavelength of 582 nm on an ELISA reader (SpectraMax M5, Molecular Devices Switzerland). Data were normalized to the amount of DNA in each culture condition, at each time point.

### 2.10 DNA content

Samples were digested overnight at 60°C with 3.9 U/mL papain from *Papaya latex* (Sigma‐Aldrich). DNA was measured by a bisbenzimide fluorescent dye (Hoechst 3258; Sigma‐Aldrich) at 350 nm excitation and 450 nm emission wavelength using a SpectraMax M5 plate reader. A standard curve from calf thymus DNA (Sigma‐Aldrich) was used. Data were normalized to the amount of cells that were seeded in each culture condition.

### 2.11 GAG content

The same papain digested samples were used to determine the amount of GAG and proteoglycans. For this, 1,9‐dimethyl‐methylene blue (Sigma‐Aldrich) was used, and absorbance was read at 600 nm with a SpectraMax M5 plate reader.^29^ GAG content was calculated from a standard curve obtained from chondroitin sulfate (Sigma‐Aldrich). Data were normalized to the amount of cells that were seeded in each culture condition.

### 2.12 Statistical analysis

Data are presented as mean ± SD (SD). For all data, a nonparametric distribution was assumed. The percentage of cells positive after cytometry analysis was analyzed by Kruskal‐Wallis tests and Dunn's multiple comparisons tests. Alizarin Red, Oil‐Red‐O, GAG, and Alamar Blue quantification were analyzed by Kruskal‐Wallis tests and Dunn's multiple comparisons tests. The relative gene expression was analyzed by two‐way ANOVA followed by a Dunnett multiple comparison test. A *P*‐value <.05 was considered significant. Asterisks within figures denote degree of statistical relevance observed: * <.05; ** <.01; ***< .001; ****< .0001. The analysis was performed using GraphPad Prism (version 8.0, GraphPad Prism Software, Inc.).

## 3 RESULTS

### 3.1 Conventional 2D monolayer culture vs 3D culture into alginate beads

**FIGURE 1:**
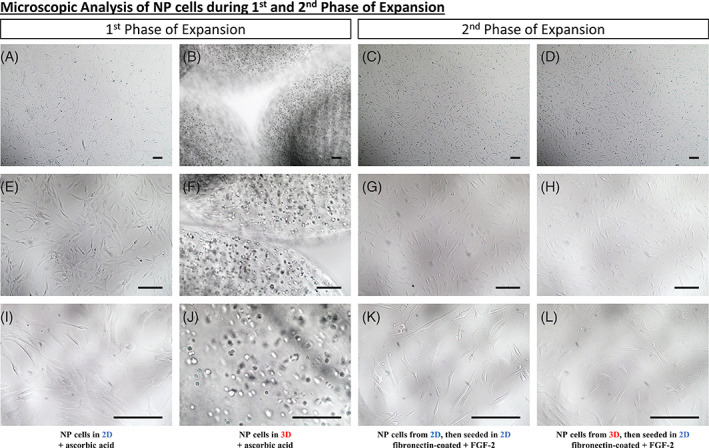
Microscopic analysis of NP cells during the first and second phase of Expansion. A‐L, Microscopic analysis of human NP cells seeded within different conditions. A, E, and I, Human NP cells seeded in two‐dimensional (2D) with a culture medium supplemented with ascorbic acid. B, F, J, Human NP cells seeded in three‐dimensional (3D) (alginate beads) with a culture medium supplemented in ascorbic acid. C, G, K, Human NP cells seeded 2D and then in 2D on fibronectin‐coated flasks with fibroblast growth factor (FGF‐2) supplemented culture medium. D, H, L, Human NP cells seeded 3D and then in 2D on fibronectin‐coated flasks with FGF‐2 supplemented culture medium. Scale bar: For pictures, A, until, L, the scale bar represents 150 μm

**FIGURE 2:**
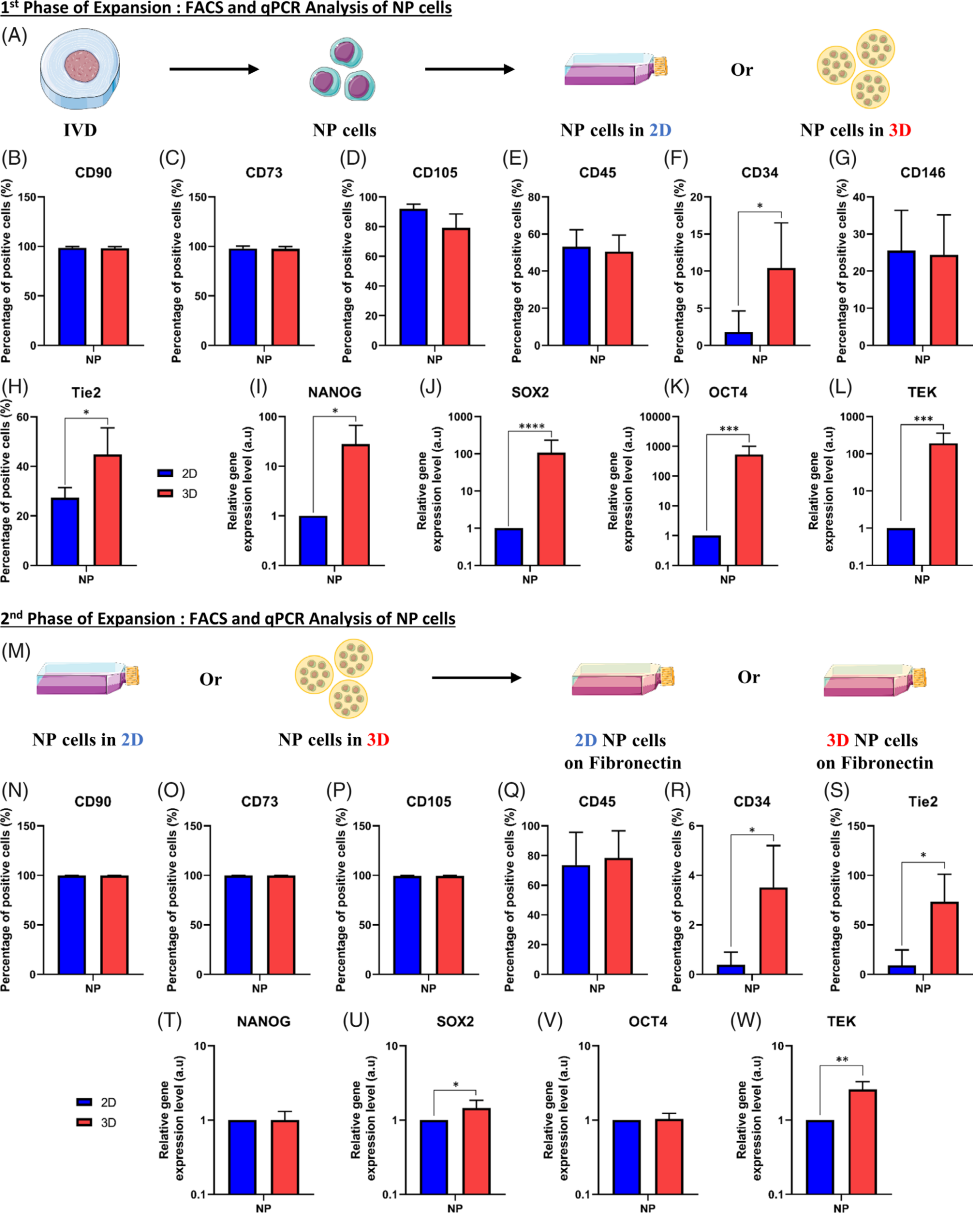
Analysis of NP cells after the first and second phase of expansion. A, Schematic representation of the first phase of expansion of nucleus pulposus (NP) cells. Briefly, after dissection and digestion of the NP tissue from the intervertebral disk (IVD), NP cells were seeded in two‐dimensional (2D) or three‐dimensional (3D) into alginate beads. B‐H, Quantification by flow cytometry analysis of the amount of NP cells positive for different markers after culture in 2D or 3D for 1 week of culture. B‐H, Percentage of NP cells positive in 2D or 3D for, B, CD90 marker, C, CD73 marker, D, CD105 marker, E, CD45 marker, F, CD34 marker, G, CD146 marker, and, H, Tie2 marker. I‐L, Quantification of the relative gene on NP cells for different genes after culture in 2D or 3D for 1 week of culture. I, Relative gene expression of *NANOG* in NP cells in 2D or 3D. J, Relative gene expression of *SOX2* in NP cells in 2D or 3D. K, Relative gene expression of *OCT4* in NP cells in 2D or 3D. L, Relative gene expression of *TEK* in NP cells in 2D or 3D. M, Schematic representation of the second phase of expansion of NP cells. Briefly, after the first phase of expansion, NP cells cultured in 2D or 3D were putting back in the fibronectin‐coated flask/surface. N‐S, Quantification by flow cytometry analysis of the amount of NP cells (previously cultivated in 2D or 3D) positive for different markers after culture in the fibronectin‐coated surface for 1 week of culture for, N, CD90 marker, O, CD73 marker, P, CD105 marker, Q, CD45 marker, R, CD34 marker, and, S, Tie2 marker. T‐W, Quantification of the relative gene on NP cells (previously cultivated in 2D or 3D) for different genes after culture on the fibronectin‐coated surface. T, Relative gene expression of *NANOG* in NP cells. U, Relative gene expression of *SOX2* in NP cells. V, Relative gene expression of *OCT4* in NP cells. W, Relative gene expression of *TEK* in NP cells (n = 6) for each gene. Data are presented as mean ± SD. NP cells cultured in 2D during the first expansion phase = blue bars. NP cells cultured in 3D during the first expansion phase = red bars. Tie2, angiopoietin‐1 receptor; *NANOG*, homeobox protein NANOG; *SOX2*, SRY (sex‐determining region Y)‐box 2; *OCT4*, octamer‐binding transcription factor 4; *TEK*, angiopoietin‐1 receptor

To find a better way to culture NPCs, we were following a two‐step protocol. Microscopic pictures of the NPCs were taken at each round of this two‐step protocol (Figure 1). Briefly, during the first phase of expansion, we observed that NPCs formed a monolayer when culturing them in 2D with ascorbic acid supplemented medium (Figure 1A,E,I). However, if cells were within alginate beads with ascorbic acid supplemented medium NPCs presented a round shape in the hydrogel (Figure 1B,F,J). During the second phase of expansion, NPCs that were previously cultured in 2D and then in 2D on a fibronectin‐coated surface with FGF‐2 supplemented medium presented a stromal phenotype (Figure 1C,K), as NPCs that were previously cultured in 3D and then in 2D on a fibronectin‐coated surface with FGF‐2 supplemented medium (Figure 1D,H,L). For the first step (first expansion phase), after dissecting the IVD and digesting of the nucleus pulposus (NP) tissue, NPCs were seeded in 2D as a monolayer or in 3D into alginate beads (Figure 2A).

After 1 week (to 1.5 weeks depending on the proliferation rate) of culture, NPCs cultured in 2D as a monolayer and 3D within alginate beads were analyzed by flow cytometry (Figure 2B‐H). In both conditions (2D or 3D), all NPCs were positive for CD90, CD75 and CD105 (Figure 2B‐D). In the same way, NPCs in 2D or in 3D were both expressing CD45 (50 ± 5%) (Figure 2E) and CD146 (25 ± 3%) (Figure 2G). In contrast, only cells cultured in 3D (alginate beads) were significantly positive for CD34 (10 ± 5%) (Figure 2F) and the Tie2 marker (45 ± 5%) (Figure 2H).

Concerning the relative expression of pluripotent genes and Tie2 (aka. *TEK* gene), 2D and 3D related samples were analyzed after 1 week of in vitro culture by qPCR (Figure 2I‐L). The relative gene expression of *NANOG* (Figure 2I), *SOX2* (Figure 2J), *OCT4* (Figure 2K), and Tie2 (aka. *TEK* gene) (Figure 2L) was significantly higher (27‐, 107‐, 531‐, and 193‐fold increase, respectively) in NPCs that were cultured culture into alginates beads compared to 2D culture on tissue culture flask.

### 3.2 NPCs maintained their gene expression profile and NPPCs proliferated when cultured back in 2D

For the second step (second expansion phase), following the first phase of expansion, NPCs cultured in 2D monolayer or 3D alginate beads were put back in 2D fibronectin‐coated flasks (Figure 2M).

To further increase the number of human NPCs, after the first phase of expansion (in 2D or 3D), we performed the second phase of expansion in 2D using fibronectin‐coated dishes with FGF‐2 supplemented medium. The analysis performed was the same as the one used for the first expansion phase, meaning flow cytometry (Figure 2N‐S) and qPCR (Figure 2T‐W).

Concerning the surface markers, all NPCs were positive for CD90, CD75, and CD105 (Figure 2N‐P) for both conditions (cell culture in 2D or 3D in the first expansion phase). In the same way, NPCs that were cultured in 2D or 3D both expressed CD45 (70 ± 7%) (Figure 2Q). In contrast, only cells cultured previously in 3D (alginate beads) were significantly positive for CD34 (3.5 ± 0.5%) (Figure 2R) and Tie2 marker (75 ± 20%) (Figure 2S).

Concerning the relative expression of pluripotent genes and Tie2 (Figure 2T‐W), the expression of *NANOG* (Figure 2T) and *OCT4* (Figure 2V) was similar in NPCs for both conditions (cells culture in 2D or 3D in the first expansion phase). However, the relative gene expression of *SOX2* (Figure 2U) and Tie2 (aka. *TEK* gene) (Figure 2W) was still significantly higher (1.4‐ and 2.5‐fold increase, respectively) in NPCs cultured in alginates beads compared to 2D culture on tissue culture flasks.

### 3.3 Adipogenic differentiation of NPCs

**FIGURE 3:**
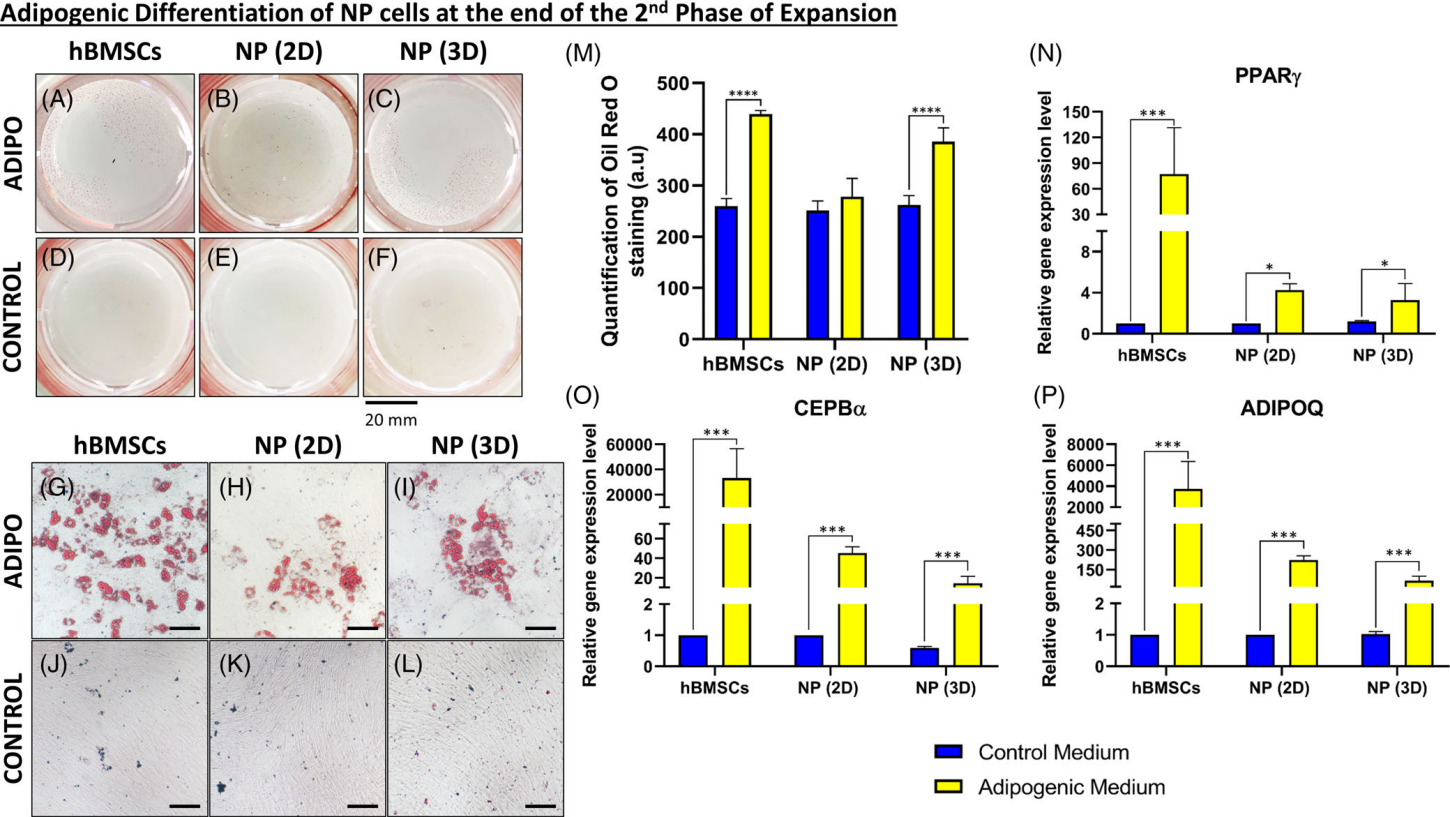
Adipogenic differentiation of NP cells at the end of the second expansion phase. A‐C, Macroscopic analysis of Oil‐Red‐O staining after 21 days of culture into the adipogenic medium for, A, human Bone marrow mesenchymal stromal cells (hBMSCs), B, nucleus pulposus (NP) cells previously cultured in two‐dimensional (2D) (NP [2D]), and, C, NP cells previously cultured in three‐dimensional (3D) (NP [3D]). D‐F, Macroscopic analysis of Oil‐Red‐O staining after 21 days of culture into control medium for, D, hBMSCs, E, NP cells previously cultured in 2D (NP [2D]), and, F, NP cells previously cultured in 3D (NP [3D]). G‐L, Microscopic analysis of Oil‐Red‐O staining after 21 days of culture into the adipogenic medium for, G, hBMSCs, H, NP cells previously cultured in 2D (NP [2D]) and, I, NP cells previously cultured in 3D (NP [3D]). J‐L, Macroscopic analysis of Oil‐Red‐O staining after 21 days of culture into control medium for, J, hBMSCs, K, NP cells previously cultured in 2D (NP [2D]), and, L, NP cells previously cultured in 3D (NP [3D]). M, Quantification of Oil‐Red‐O staining for hBMSCs, NP cells previously cultured in 2D (NP [2D]), and NP cells previously cultured in 3D (NP [3D]) after 21 days in the adipogenic and control medium. N‐P, Quantification of the relative gene on NP cells (previously cultivated in 2D or 3D) for different adipogenic related genes after culture on the fibronectin‐coated surface with the adipogenic or control medium. N, Relative gene expression of peroxisome proliferator‐activated receptor gamma (*PPARγ*) in NP cells. O, Relative gene expression of CCAAT/enhancer‐binding protein alpha (*CEPBα*) in NP cells. P, Relative gene expression of adiponectin (*ADIPOQ*) in NP cells (*n* = 6) for each gene. Data are presented as mean ± SD. NP cells cultured in the control medium = blue bars. NP cells cultured in the adipogenic medium = yellow bars. Scale bar: For pictures, G, until, L, the scale bar represent 100 μm

Next, we aimed to differentiate the expanded NPCs (from second expansion phase) into an adipogenic lineage and to compare them with hBMSCs. After 21 days of culture, Oil‐Red‐O staining was performed and a macroscopic (Figure 3A‐F) and microscopic (Figure 3G‐L) representation of this assay are shown. At a macroscopic and microscopic level, we observed stained lipid droplets (in red/pink) only with cells cultured with adipogenic medium (Figure 3A‐C and Figure 3G‐I) and no evidence with cells cultured with the control medium (Figure 3D‐F,J‐L).

After quantifying the Oil‐Red‐O stain (Figure 3M), we could observe that a higher amount was stained with hBMSCs and 3D NPCs compared to 2D NPCs (1.7‐fold increase for both).

Regarding the relative gene expression of adipogenic markers, *PPARγ* (Figure 3N), *CEPBα* (Figure 3O), and *ADIPOQ* (Figure 3P) were all highly expressed in the condition treated with adipogenic medium compared to control medium, for hBMSCs, NPCs cultured in 2D (aka. NP (2D)) and NPCs cultured in 3D (aka. NP [3D]).

### 3.4 Osteogenic differentiation of NPCs

**FIGURE 4:**
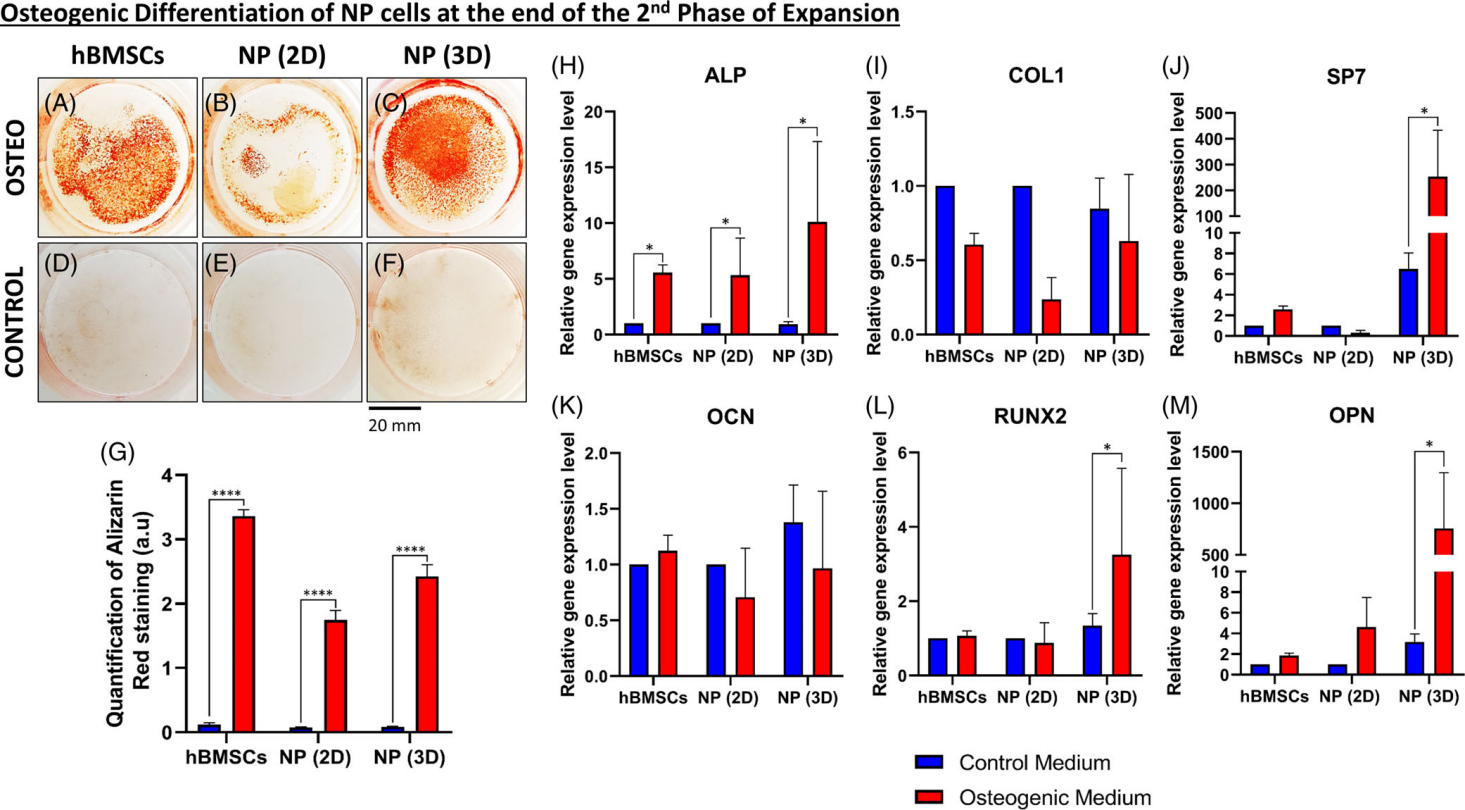
Osteogenic differentiation of NP cells at the end of the second expansion phase. A‐C, Macroscopic analysis of Alizarin Red staining after 21 days of culture into the osteogenic medium for, A, human bone marrow mesenchymal stromal cells (hBMSCs), B, nucleus pulposus (NP) cells previously cultured in two‐dimensional (2D) (NP [2D]), and, C, NP cells previously cultured in three‐dimensional (3D) (NP [3D]). D‐F, Macroscopic analysis of Alizarin Red staining after 21 days of culture into the control medium for, D, hBMSCs, E, NP cells previously cultured in 2D (NP [2D]), and, F, NP cells previously cultured in 3D (NP [3D]). G, Quantification of Alizarin Red staining for hBMSCs, NP cells previously cultured in 2D (NP [2D]), and NP cells previously cultured in 3D (NP [3D]) after 21 days in the osteogenic and control medium. H‐M, Quantification of the relative gene on NP cells (previously cultivated in 2D or 3D) for different osteogenic related genes after culture on the fibronectin‐coated surface with the osteogenic or control medium. H, Relative gene expression of alkaline phosphatase (*ALPL*) in NP cells. I, Relative gene expression of collagen type I (*COL1*) in NP cells. J, Relative gene expression of osterix (*SP7*) in NP cells. K, Relative gene expression of osteocalcin (*OCN*) in NP cells. L, Relative gene expression of runt‐related transcription factor 2 (*RUNX2*) in NP cells. M, Relative gene expression of osteopontin (*OPN*) in NP cells (n = 5) for each gene. Data are presented as mean ± SD. NP cells cultured in the control medium = blue bars. NP cells cultured in the osteogenic medium = red bars

Afterwards, we attempted to differentiate the expanded NPCs (at the end of the second expansion phase) into an osteogenic lineage compared to hBMSCs. After 21 days of culture, ALZR staining was performed and a representation of this assay is shown (Figure 4A‐F). We could observe a coloration of the mineralized extracellular matrix (in red) only in the wells containing cells cultured with osteogenic medium (Figure 4A‐C) compared to the control medium (Figure 4D‐F), for hBMSCs, NPCs cultured in 2D (aka. NP [2D]) and NPCs cultured in 3D (aka. NP [3D]).

After quantification of the ALZR stain by re‐solubilizing the coloration (Figure 4G), we could observe a significantly higher amount (at least a twofold increase) of stained cells with hBMSC, NP (2D) cell, and NP (3D) cell cultures with osteogenic medium compared to the same cells in the control medium.

Regarding the relative gene expression of osteogenic markers, we could observe an up‐regulation (between 5‐ and 10‐fold increase) of *ALPL* (Figure 4H) for hBMSCs, NP (2D) cells, and NP (3D) cells cultivated in the osteogenic medium in comparison to control medium. Concerning *COL1* (Figure 4I) and *OCN* (Figure 4K), no differences were observed. Else ways, looking at *SP7* (Figure 4J), *RUNX2* (Figure 4L), and *OPN* (Figure 4M) we could note than only the NP (3D) cells were showing a significant increase of relative gene expression of those genes (40‐, 3‐, and 187‐fold increase, respectively) in the condition treated with osteogenic medium compared to the control medium.

### 3.5 Chondrogenic differentiation of NPCs

**FIGURE 5:**
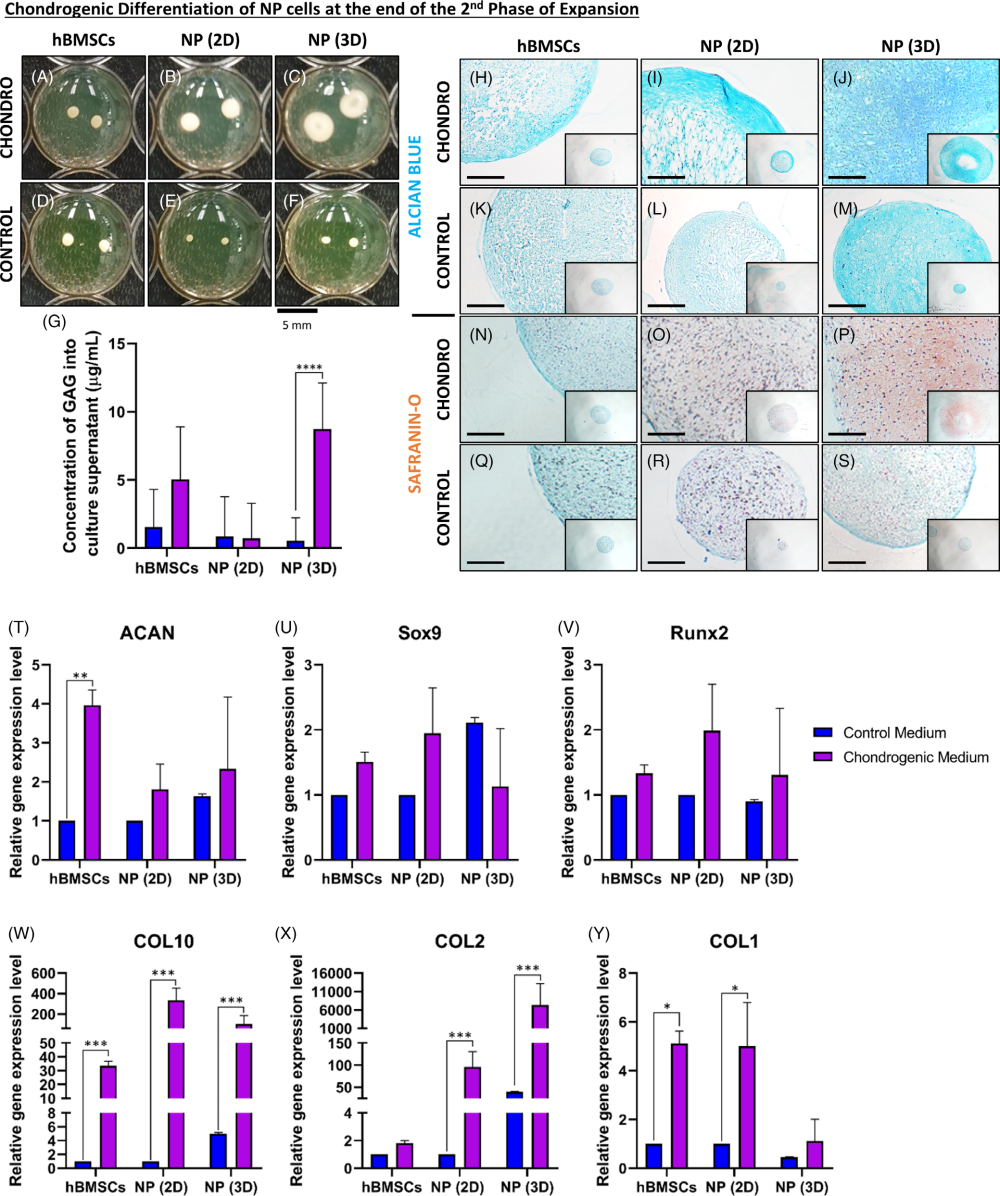
Chondrogenic differentiation of nucleus pulposus (NP) cells at the end of the second expansion phase. A‐C, Macroscopic analysis of samples fixed after 21 days of culture into chondrogenic medium for, A, human bone marrow mesenchymal stromal cells (hBMSCs), B, NP cells previously cultured in two‐dimensional (2D) (NP [2D]), and, C, NP cells previously cultured in three‐dimensional (3D) (NP [3D]). D‐F, Macroscopic analysis of samples fixed after 21 days of culture into control medium for, D, hBMSCs, E, NP cells previously cultured in 2D (NP [2D]), and, F, NP cells previously cultured in 3D (NP [3D]). G, Quantification of GAG into culture supernatant for hBMSCs, NP cells previously cultured in 2D (NP [2D]), and NP cells previously cultured in 3D (NP [3D]) after 21 days in chondrogenic and control Medium. H‐M, Microscopic analysis of Alcian Blue staining after 21 days of culture into chondrogenic medium for, H, hBMSCs, I, NP cells previously cultured in 2D (NP [2D]) and, J, NP cells previously cultured in 3D (NP [3D]). K‐M, Macroscopic analysis of Alcian Blue staining after 21 days of culture into control medium for, K, hBMSCs, L, NP cells previously cultured in 2D (NP [2D]), and, M, NP cells previously cultured in 3D (NP [3D]). N‐P, Microscopic analysis of Safranin‐O and Fast Green staining after 21 days of culture into chondrogenic medium for, N, hBMSCs, O, NP cells previously cultured in 2D (NP [2D]), and P, NP cells previously cultured in 3D (NP [3D]). Q‐S, Macroscopic analysis of Safranin‐O and Fast Green staining after 21 days of culture into control medium for, Q, hBMSCs, R, NP cells previously cultured in 2D (NP [2D]) and, S, NP cells previously cultured in 3D (NP [3D]). T‐Y, Quantification of the relative gene on NP cells (previously cultivated in 2D or 3D) for different chondrogenic‐related genes after culture on the fibronectin‐coated surface with chondrogenic or control medium. T, Relative gene expression of aggrecan (*ACAN*) in NP cells. U, Relative gene expression of transcription factor Sox‐9 (*SOX9*) in NP cells. V, Relative gene expression of runt‐related transcription factor 2 (*RUNX2*) in NP cells. W, Relative gene expression of collagen type X (*COL10*) in NP cells. X, Relative gene expression of collagen type II (*COL2*) in NP cells. Y, Relative gene expression of collagen type I (*COL1*) in NP cells (*n* = 5) for each gene. Data are presented as mean ± SD. NP cells cultured in the control medium = blue bars. NP cells cultured in the chondrogenic medium = violet bars. Scale bar: For pictures, H, until, S, the scale bar represents 500 μm

Subsequently, we tried to differentiate the expanded NPCs (at the end of the second expansion phase) toward a chondrogenic lineage and compared the results with hBMSCs. After 21 days of culture, samples were fixed and analyzed macroscopically (Figure 5A‐F). After 21 days, cell pellets cultured in the chondrogenic medium (Figure 5A‐C) were bigger and whiter than cells cultured in the control medium (Figure 5D‐F). Thereafter, we also quantified the GAG content in the supernatant (Figure 5G). We showed that the amount of GAG was significantly higher (10 μg/mL compared to 0.5 μg/mL) in the NP (3D) cells cultured with chondrogenic medium compared to the control medium (Figure 5G).

To continue to analyze those samples, two histological colorations were performed: Alcian Blue (Figure 5H‐M) and Safranin‐O coupled with Fast Green (Figure 5N‐S), and sections were analyzed microscopically. For the Alcian Blue staining, we could observe a stronger intensity of blue stain, showing the presence of proteoglycan components, for the constructs that were cultured in the chondrogenic medium (Figure 5H‐J) compared to the same cells cultured in the control medium (Figure 5K‐M). Concerning the Safranin‐O staining, we noticed a stronger intensity of red/orange stain, showing the presence of GAG, for the constructs that were cultured in the chondrogenic medium (Figure 5N‐P) compared to the same cells cultured in the control medium (Figure 5Q‐S). Moreover, as described above, the size of the constructs/cell pellets, especially in the NP (3D) cell conditions cultured with the chondrogenic medium were bigger and were presenting a “donut” shape (Figure 5J,P).

Concerning the relative gene expression of chondrogenic markers, we saw an up‐regulation (fourfold increase) of *ACAN* (Figure 5T) only for hBMSCs cultivated in the chondrogenic medium in comparison to the control medium. Regarding *SOX9* (Figure 5U) and *RUNX2* (Figure 5V), no differences were observed in any conditions. To continue, *COL10* (Figure 5W), a marker of hypertrophic differentiation, we could see an up‐regulation for all conditions compared to hBMSCs cultured in control medium, NP (2D) cells, and NP (3D) cells (30, 300 and 200‐fold increase, respectively). Looking at *COL2* (Figure 5X) only NPCs cultured in 2D or 3D was showing an up‐regulation (78 and 8000‐fold increase, respectively) compared to the control medium condition. For *COL1* (Figure 5Y) an up‐regulation for hBMSCs and 2D NPCs (fivefold increase, for both) was found in the condition treated with chondrogenic medium compared to the control medium.

### 3.6 Expression of *TEK* gene (aka. Tie2) after differentiation

**FIGURE 6:**
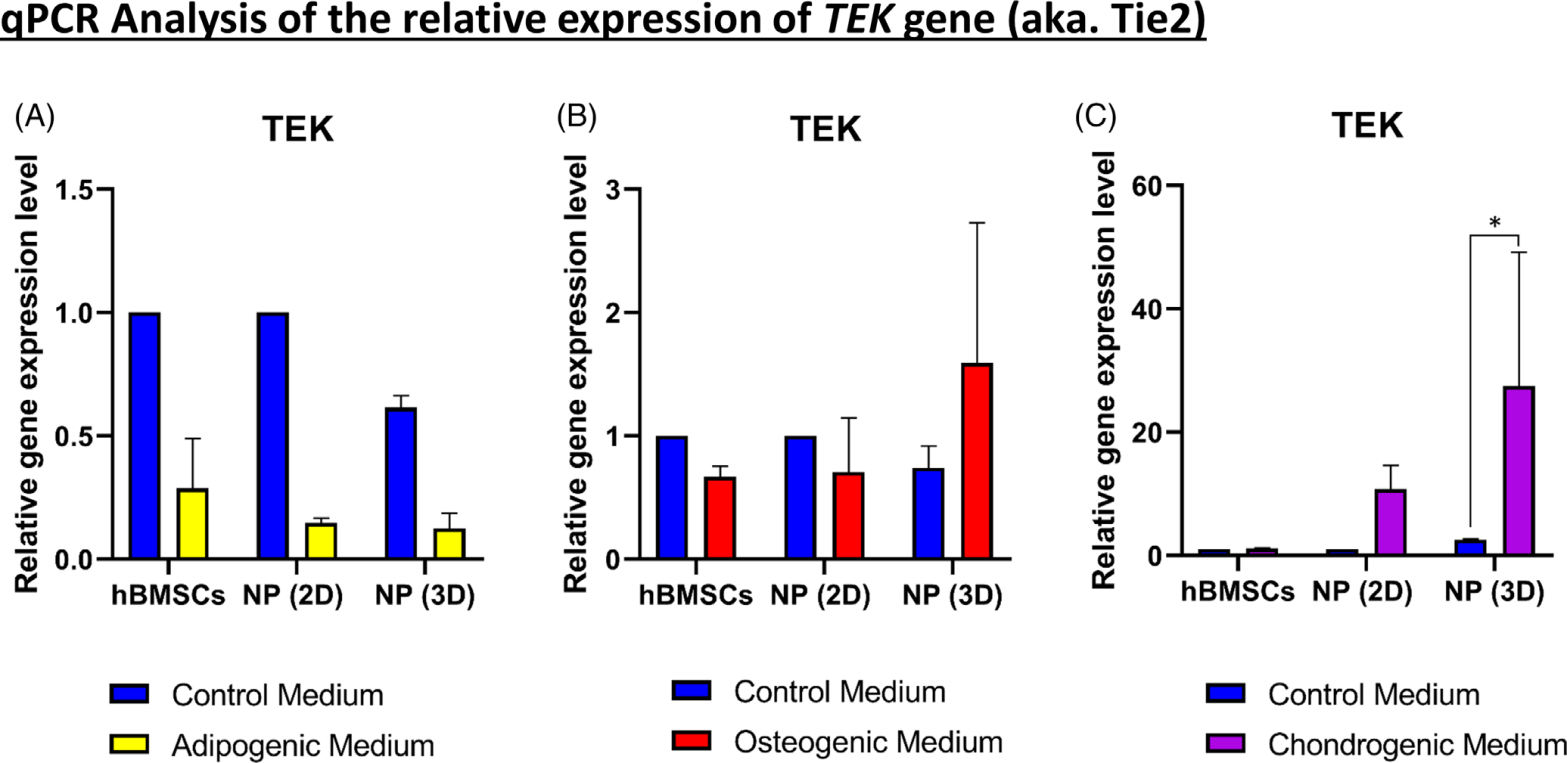
Expression of angiopoietin‐1 receptor (*TEK*) gene after 21 days of tri‐lineage differentiation. A,B, Quantification of the relative gene on nucleus pulposus (NP) cells (previously cultivated in two‐dimensional [2D] or three‐dimensional [3D]) for *TEK* gene after tri‐lineage differentiation. A, Relative gene expression of *TEK* in human bone marrow mesenchymal stromal cells (hBMSCs) and NP cells cultivated in the adipogenic medium. B, Relative gene expression of *TEK* in hBMSCs and NP cells cultivated in the osteogenic medium. C, Relative gene expression of *TEK* in hBMSCs and NP cells cultivated in the chondrogenic medium (*n* = 5) for each differentiation medium. Data are presented as mean ± SD. NP cells cultured in control medium = blue bars. NP cells cultured in the adipogenic medium = yellow bars. NP cells cultured in the osteogenic medium = red bars. NP cells cultured in the chondrogenic medium = violet bars

To explore the behavior of Tie2^+^ cells, the relative gene expression of *TEK* was quantified after 21 days of adipogenic (Figure 6A), osteogenic (Figure 6B), and chondrogenic (Figure 6C) differentiation of hBMSCs, NP (2D) cells, and NP (3D) cells. No significant up‐ or down‐regulation was observed concerning adipogenic (Figure 6A) and osteogenic (Figure 6B) differentiation for hBMSCs, NP (2D) cells or 3D NP (3D) cells. In contrast, after 21 days of culture, we noticed a significant up‐regulation (26‐fold increase) of the *TEK* gene (Tie2) only for NP (3D) cells cultured with chondrogenic medium compared to ones cultured with the control medium (Figure 6C).

### 3.7 Comparison of Tie2^+^ and Tie2^−^ NP cells population

**FIGURE 7:**
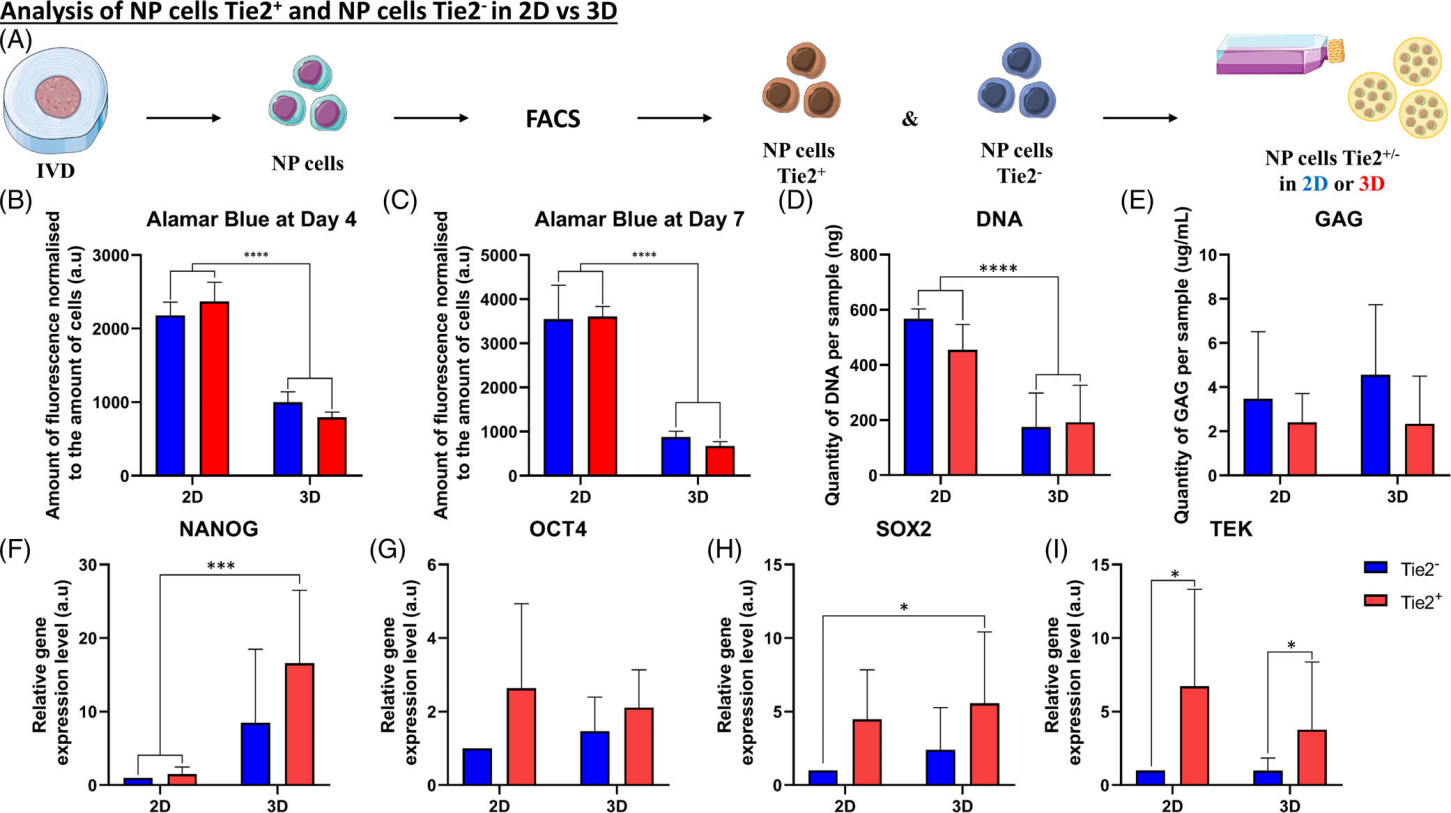
Comparison of nucleus pulposus (NP) cells Tie2^+^ and NP cells Tie2^−^. A, Schematic representation of the process leading to the comparison of NP cells Tie2^+^ and NP cells Tie2^−^. Briefly, after dissection and digestion of the NP tissue from intervertebral disk (IVD), NP cells were sorted for angiopoietin‐1 receptor (Tie2) marker. Positive (Tie2^+^) and negative (Tie2^−^) cells were seeded in two‐dimensional (2D) or in three‐dimensional (3D) (into alginate beads) and analyzed. A,B, Quantification of cell metabolism by Alamar Blue assay on Tie2^+^ and Tie2^−^ NP cells (cultivated in 2D or 3D) at, A, day 4 and, B, day 7. D, Quantification of GAG produced in NP cells (Tie2^+^ and Tie2^−^) in 2D or in 3D at day 7. E, Quantification of DNA measured in NP cells (Tie2^+^ and Tie2^−^) in 2D or in 3D after day 7. F‐I, Quantification of the relative gene expression on NP cells (Tie2^+^ and Tie2^−^) after culture in 2D or in 3D for 1 week of culture. F, Relative gene expression of homeobox protein NANOG (*NANOG*) in NP cells (Tie2^+^ and Tie2^−^) in 2D or in 3D. G, Relative gene expression of SRY (sex determining region Y)‐box 2 (*SOX2*) in NP cells (Tie2^+^ and Tie2^−^) in 2D or in 3D. H, Relative gene expression of octamer‐binding transcription factor 4 (*OCT4*) in NP cells (Tie2^+^ and Tie2^−^) in 2D or in 3D. I, Relative gene expression of angiopoietin‐1 receptor (*TEK*) in NP cells (Tie2^+^ and Tie2^−^) in 2D or in 3D (*n* = 5) for each gene. Data are presented as mean ± SD. Culture of NP cells (Tie2^−^) = blue bars. Culture of NP cells (Tie2^+^) = red bars

To look at the effect of Tie2^+^ cells on the expression of pluripotent genes and other factors, we followed the two‐step protocol previously described with the addition of a sorting step (FACS) after digesting the NP tissue (Figure 7A). Briefly, after dissecting the human IVD and digesting the NP tissue, NPCs were sorted for the Tie2 marker. Next, Tie2^+^ and Tie2^−^ NP cells were seeded in 2D as a monolayer or 3D into alginate beads (Figure 7A).

We initially looked at the cell metabolism using the Alamar Blue assay on day 4 (Figure 7B) and day 7 (Figure 7C), normalized to the amount of DNA. We noticed that the cell metabolism (proportional to the amount of DNA) was lower in cells (either Tie2^+^ or Tie2^−^) cultured in 3D compared to cells cultured in 2D on day 4 and day 7 (twofold and 3.5‐fold decrease, respectively). Moreover, looking at the DNA content at day 7 (Figure 7D), in each condition, we can observe a lower amount of DNA measured in 3D culture (either Tie2^+^ or Tie2^−^) compared to 2D culture (2.25‐fold decrease). Concerning the GAG load of each sample at day 7 (Figure 7E), we can note no statistical differences between each condition (either Tie2^+^ or Tie2^−^ and 2D or 3D culture).

Regarding the relative expression of pluripotent genes and Tie2 (Figure 7F‐I), the expression of *NANOG* (Figure 7F) and *SOX2* (Figure 7H) was showing a significant up‐regulation for Tie2^+^ NPCs that were cultured in 3D compared to NPCs cultured in 2D (either Tie2^+^ or Tie2^−^). However, *OCT4* (Figure 7G) was not showing any statistical differences in NPCs for both conditions, so for cells cultured in 2D or 3D (either Tie2^+^ or Tie2^−^). Finally, looking at the relative gene expression of Tie2 (aka. *TEK* gene) (Figure 7I), we can observe that Tie2^+^ NPCs (either in 2D or 3D culture) were expressing more *TEK* gene than to Tie2^−^ NPCs (7.2‐ and 4.1‐fold increase, for 2D and 3D culture, respectively).

## 4 DISCUSSION

In this study, we established and studied a novel and efficient two‐phase expansion culture method for human NPCs, using a series of primary 3D alginate bead culture followed by 2D monolayer culture on fibronectin‐coated dishes with FGF‐2.

In the first phase of expansion, NPCs cultured in alginate beads (3D) showed the most relevant results. First, NPCs cultured in 3D were showing the same phenotype as hBMSCs by expressing CD90, CD73, CD105, and CD45 as surface markers.^30^ Moreover, cells cultured in 3D presented a higher percentage of cells positive for CD34 (10 ± 6.2%) compared to NPCs cultured in 2D (2.5 ± 2.3%) showing a higher potential of differentiation of this population (3D) compared to the 2D as CD34 is a common marker for diverse progenitors.^31^ Moreover, the expression of the CD34 surface marker by a small percentage of NPCs could also suppose that those cells are closely related to quiescent hematopoietic stem cells/progenitor cells that were identified to express both CD34 and Tie2.^32^, ^33^, ^34^ Only CD146 did not reveal any differences in our analysis. This could be explained by the fact that disks were coming from a healthy donor and that this marker can distinguish stem cell subpopulations with distinct migration and regenerative potential in degenerative IVDs.^35^ Concerning the expression of Tie2, already at the end of the first phase of expansion, the percentage of cells expressing this NPPCs characteristic marker,^7^ was much higher when cells were cultured in alginate beads than the 2D monolayer on plastic. This observation was showing that the 3D culture was better mimicking the microenvironment of the NP tissue and keeping NPCs in a more native surrounding inducing a higher percentage of Tie2 expressing cells.

Furthermore, in a matter of keeping the gene expression of pluripotent genes like *NANOG*, *SOX2*, and *OCT4*, or by keeping the expression of the *TEK* gene, the 3D configuration of NPCs was far better than the 2D culture. Those results can be compared with the recent use of the limiting dilution method for the isolation of rat NP mesenchymal stem/progenitor cells (NPMSCs) and the effect achieved in vitro on various NPMSCs biological characteristics, especially cell activity and plasticity.^36^

All the results and observations related to the 3D culture into alginate beads can be possibly attributable to the fact that human NP cells are surrounded in their native tissue by ECM and connective tissues in vivo and may, therefore, favor environments in which they are less stressed (in particular by integrin pathway). The 3D culture used in this study maintained the NP phenotype, as confirmed by the expression patterns of surface markers (Tie2 and CD34) and genes (*NANOG*, *OCT4*, *SOX2*, and *TEK*).

Regarding the second phase of expansion, the addition of FGF‐2 accelerated the proliferation of human NPCs (data not shown), as already described.^37^ This result is consistent with previous studies, which have reported enhanced cell proliferation following FGF‐2 administration.^16^, ^38^, ^39^ However, in agreement with previous reports,^40^, ^41^ the culture of NPCs in monolayer (2D) with or without FGF‐2 leads to a loss of the phenotype. Moreover, fibronectin has been shown to be a key component in IVDs and more precisely NP tissue.^42^, ^43^, ^44^ In this study, the combination of fibronectin coating with the addition of FGF‐2 into the culture media, as previously described with the culture of murine NPCs, should overcome this limitation of “stemness” potential loss.^45^ This expansion protocol, by still keeping the cells in a mimicking microenvironment after previously cultured in 3D (alginate beads), will maintain the cells with a phenotype and gene expression close to cells in their native tissue. Briefly, even at the end of the second phase of expansion, NPCs were still showing a phenotype similar to hBMSCs, meaning CD90^+^, CD73^+^, CD105^+^, and CD45^+^. Moreover, only cells previously cultured in 3D (in the first phase) were still positive for CD34 and Tie2, two characteristic markers of NPPCs of the IVD across species.^9^ However, the fibronectin coating combined with the treatment with FGF‐2 was not as efficient as the 3D culture in alginate, which can be seen with a lower expression of two pluripotent genes, *NANOG* and *OCT4*, but still a higher relative gene expression of *SOX2* and *TEK* for the cells previously cultured into alginate beads. All those genes are well described for the regulation of stem cell pluripotency and differentiation^46^ but are also directly described and involved in induced pluripotent stem cells from human nucleus pulposus cells.^47^

Each culture attempt (first and second expansion phase) was successful in every trial. Despite the small number of human NPCs initially extracted after NP tissue digestion and subsequent seeding into 3D alginate beads, successful proliferation and meaningful increase of the Tie2 expressing population was achieved. The investigation of other markers reflecting the physiological behavior of NPCs, like the quantification of HIF‐1α, Collagen I/Collagen II ratio, or Aggrecan/Collagen type II ratio, could have been also done to improve the quality of our study, however, due to the early time point of the analysis, it was not possible to consider them. This lack of characterization can be considered as a limitation for our study.

To verify the multipotent potential of the NPCs that we were expanding, we decided to perform a tri‐lineage differentiation of those cells into adipogenic, osteogenic, and chondrogenic differentiation as previously described.^26^, ^48^ Osteogenic and adipogenic differentiation of NPCs were performed at 20% oxygen as difficulties to obtain mineralization and adipogenesis at low oxygen levels are well documented in the literature.^49^, ^50^, ^51^, ^52^, ^53^, ^54^ Culturing in adipogenic medium following a second phase expansion culture with FGF‐2 was showing a great formation of lipid droplets in our control condition (hBMSCs) but also in both NPCs samples (2D and 3D). This was already described in the literature^55^ and confirmed the stem potential of our expanded cells. Concerning the osteogenic potential after 21 days of culture, the NPCs (2D and 3D) also showed a strong production of extracellular mineralized matrix, comparable to the hBMSCs. This osteogenic potential was already described,^56^ however, human NPCs can become osteogenic in monolayer, and calcification of the extracellular matrix was occurring consistently.

In previous studies, it has been reported that culturing cells in chondrogenic medium with subsequent culture with FGF‐2 increases chondrocyte‐specific gene expression and ECM synthesis.^57^, ^58^, ^59^ In particular, Tekari et al. showed that Tie2 expression in bovine NPCs population decreased from approximately 8% to less than 1% after 2.3 population doublings.^8^ However, Tie2^+^ preservation could be augmented under hypoxic conditions, by supplementation of FGF‐2 or synergistic FGF‐2 and hypoxic conditions.^9^ In this study, human NP cells showed the appearance of “donut” like constructs after 21 days only if cells were cultured previously into 3D alginate beads before differentiation. Therefore, the in vitro culture into the chondrogenic medium for pellets and micro mass in this study were able to maintain the gene expression of *TEK* (Tie2) only if cells were cultured previously into 3D alginate beads prior to differentiation. These conditions might contribute to the recovery of NPPCs even after the formation of the NP chondrogenic pellets that we produced in vitro.

Nonetheless, the addition of TGF‐β3 to the chondrogenic medium was enhancing the production of *COL10*, a marker of hypertrophic differentiation of chondrocytes. However, the relative expression of RUNX2 was still at a relatively low level, supposing that our NP chondrogenic pellets were potentially not reaching the stage of matrix mineralization.

Besides, we used TGF‐β3 supplemented medium during NPC culture for chondrogenic differentiation instead of TGF‐β1. This resulted in a high expression of *COL10* at the end of the 21 days of differentiation. However, the NP chondrogenic pellets stemming from NP cells cultured in 3D (alginate beads) still showed a high amount of *COL2* and *ACAN*, meaning that the hypertrophic stage was not too advanced. Further research is needed to optimize the combined use of growth factors required for the differentiation of NPC phenotypes.

Interestingly, the NPCs' population enriched in Tie2^+^ cells used here (ie, the population with the highest percentage of Tie2^+^), had a superior differentiation capacity towards the chondrogenic lineage compared to hBMCs from the same patients as described previously with NPCs extracted from degenerated IVD.^60^

This study has several limitations. First, we did not distinguish between “notochordal cells” and “chondrocyte‐like cells” among our mix of NP cells after dissection and digestion of the NP tissue.^6^, ^61^ However, morphological differences between “notochordal” cells and “chondrocyte‐like”/“nucleopulpocyte‐like” cells have recently been distinguished in the stages of cellular differentiation.^6^, ^62^, ^63^, ^64^, ^65^, ^66^, ^67^ In this study, both large vacuolated cells and small round cells were observed during the early stages of 3D alginate bead cultures (Figure 1). However, the small cells dominated the cell population as cell proliferation increased. Accordingly, NP “chondrocyte‐like” and NP progenitor cells identified as small cells were mainly used in this study.

Regarding the Tie2^+^ and Tie2^−^ experiment, the first phase of expansion with 3D culture (alginate beads) seems to keep the NPCs (either Tie2^+^ or Tie2^−^) at a lower metabolism, compared to 2D culture, by having them in a microenvironment close to the native NP tissue.^68^, ^69^ Increasing evidence highlights a pivotal role for metabolism in stem cell physiology and lineage specification.^70^, ^71^ Metabolism, indeed, is no longer considered merely an energy source nor an endpoint of gene regulation. Instead, metabolites and the nutrient environment are active players in determining intracellular signaling and enzymatic activities and consequently modulators of the stem cell's fate. Moreover, metabolic intermediates of cellular metabolism regulate epigenetic mechanisms, including histone modifications, DNA methylation, and noncoding RNAs, thereby modulating the global epigenome landscape and stemness.^72^ Concerning GAG production by NPCs (either Tie2^+^ or Tie2^−^), it was impossible to detect any statistical difference probably due to the early time point of measurement (day 7), knowing that it was early shown that ECM mimicking scaffold (including GAG) promote stemness maintenance of mesenchymal stem cells via spheroid formation.^73^ Recent research showed that the enrichment of the culture environment, either by chemically defined medium, growth factor supplementation, or modification of the culture surface stiffness, helped to preserve progenitor cells.^74^, ^75^

Concerning the pluripotent gene expression for NP Tie2^+^ and Tie2^−^ cells, the higher expression of *NANOG* and *SOX2* only found in NPCs positive for Tie2 and cultured in 3D reveals the central role of 3D mimicking microenvironment for NPPCs culture. Even if in 2D, during the first phase of expansion Tie2^+^ cells were still expressing the *TEK* gene (aka. Tie2), they were however not able to maintain any of their pluripotent gene expression, as described with hBMSCs.^76^

Our method has the potential to become a useful tool for basic research and clinical research (cell therapy) aiming at elucidating molecular mechanisms in human NPCs and in developing cell‐based regenerative therapies for IVD degeneration using expanded NPCs population enriched in NPPCs (Tie2^+^), which cannot be obtained using a conventional monolayer culture method.^77^ Furthermore, it will be useful for research related to tissue engineering or regenerative medicine in which the expansion of progenitor cells plays an important role.

## 5 CONCLUSIONS

We established a novel and efficient primary culture and expansion culture method for human NPCs consisting of sequential primary 3D alginate culture with ascorbic acid supplemented medium followed by 2D monolayer culture on fibronectin‐coated dishes containing FGF‐2. This protocol shows that heterogenic NP cell populations are closer to a multipotent phenotype if cultured in 3D within alginate beads compared to 2D culture. Moreover, NPCs were able to better differentiate into osteogenic, chondrogenic, and to a lesser extent adipogenic lineage even after in vitro expansion than 2D monolayer expanded NPCs. As we experimented with pure Tie2^+^ and Tie2^−^ cell populations, it is highly suggested that the maintenance of multipotent capacity was mainly but not exclusively due to the higher presence of Tie2^+^ cells in the 3D culture. This project not only has a scientific impact by evaluating the influence of a two‐step expansion protocol on the functionality of NP progenitors but could also lead to an innovative clinical approach with cell therapy for IVD regeneration and repair.

## CONFLICT OF INTEREST

The authors indicated no potential conflict of interest.

## AUTHOR CONTRIBUTIONS

Julien Guerrero: Study conception and design, acquisition of main data, analysis, and interpretation of data, drafting of the main manuscript. Sonja Häckel: Acquisition of data, surgical sample contribution. Andreas Shaun Croft: Acquisition of data, editing of the manuscript. Christoph Emanuel Albers: Drafting and editing of the manuscript, and acquisition of funding. Benjamin Gantenbein: Study conception, design, interpretation of data, drafting and editing of the manuscript, and acquisition of funding.
